# A Case Report of Mandibular Gingival Metastasis From Sigmoid Colon Adenocarcinoma With Phenotypical Transformation Into Neuroendocrine Carcinoma

**DOI:** 10.7759/cureus.62805

**Published:** 2024-06-21

**Authors:** Yanko G Yankov, Ralitsa V Yotsova, Lyuben Stoev, Nikolay I Nikolaev, Simeon Dimanov, Martina Stoeva

**Affiliations:** 1 Clinic of Maxillofacial Surgery, University Hospital "St. Marina", Varna, BGR; 2 Department of General and Operative Surgery, Medical University "Prof. Dr. Paraskev Stoyanov", Varna, BGR; 3 Department of Oral Surgery, Medical University of Varna, Varna, BGR; 4 Department of General and Clinical Pathology, Forensic Medicine and Deontology, Medical University of Varna, Varna, BGR

**Keywords:** hysterectomy, sigmoid colon, colon, gingiva, oral surgery, resection, mandible, metastasis, adenocarcinoma, maxillofacial surgery

## Abstract

This case report presents a 59-year-old female patient with mandibular gingival metastasis from adenocarcinoma of the sigmoid part of the colon, who underwent radical colectomy with simultaneous hysterectomy involving and left oophorectomy (due to tumor involvement) eight years ago. Because of metastatic spread to the liver, a partial left lateral lobectomy was performed, and because of a metastatic lesion in the left adrenal gland, the latter was excised and a partial resection of the left kidney was performed. The patient was given a number of courses of chemotherapy, target therapy, and immunotherapy. In 2024, because of a tumor mass in the oral cavity that was growing and interfering with normal nutrition and speech, she was hospitalized and a radical resection of the lesion was performed along with the involved underlying bone of the lower jaw on the right. The morphological analysis revealed metastasis from large cell neuroendocrine carcinoma and the immunohistochemical stains verified the gastrointestinal origin of the lesion. The lesion was accepted as being a result of the phenotypical transformation of the primary adenocarcinoma of the sigmoid colon. The patient had a normal postoperative period and a smoothly healing wound and continued to be under the management of clinical oncologists supporting chemo-, targeted, and immunotherapy. However, five months after the appearance of the lesion and three months after its surgical removal, after a serious deterioration of her general condition, she passed away at home.

## Introduction

Metastatic disease is the spread of cancer cells by a number of mechanisms from the primary site to distant tissues or organs. Their clinical presentation can be highly variable and can present a serious diagnostic challenge for the clinician [1]. Metastatic tumors in the oral cavity are rare and constitute less than 1% of the malignant tumors [2]. Patients between 50 and 70 years of age are most affected, with no significant differences in the incidence between genders [3].

The human jaws are affected twice as often by metastatic tumors compared to the oral mucosa [4]. As for the upper and lower jaws, the mandible is more often involved, and of the soft tissues, the gingiva is first in frequency [5,6]. In soft-tissue localization, the attached gingiva is affected in 60% of cases, and the tongue in 18% [2]. When localized in the gingival area, they are most often located in the area of ​​the molars (about 50%), then in the premolars area (about 38%), and at the angle of the lower jaw (29%). Metastases most often affect the area of ​​an extracted tooth and have the appearance of a soft tissue mass that can cause discomfort and pain. The metastatic tumors can occur even without a tooth being extracted, and then they are most often in the area of ​​the attached gingiva, around the tooth, and often lead to the appearance of pathological mobility of the latter [2].

The site where the metastatic tumor develops depends on the location of the primary tumor [7]. Histologically, about 70% of metastatic tumors in the oral cavity are adenocarcinomas [8]. All malignant tumors can metastasize to the oral cavity, but most often the primary tumor involves the lungs, kidneys, liver, and prostate gland in male patients and mammary glands, genitals, and kidneys in female patients [5]. Metastases in the maxillofacial region and the oral cavity are often multiple and are associated with a high mortality rate [9,10].

Large cell neuroendocrine carcinoma (LCNEC) is a rare and aggressive tumor that occurs most commonly in the lungs with an incidence of 2-4% of all lung carcinomas. Other primary sites include the gastrointestinal tract, uterus, prostate, and kidneys [11]. Colorectal neoplasms metastasize most often to regional lymph nodes, the liver, and lungs [11]. Oral metastases are rare and have an extremely poor prognosis with a survival of about nine months after diagnosis [8]. This is exactly the clinical case that we present in the current case report, the purpose of which is to share our experience with this pathology and provide up-to-date and valuable information on the topic to fellow clinicians.

## Case presentation

**Figure 1:**
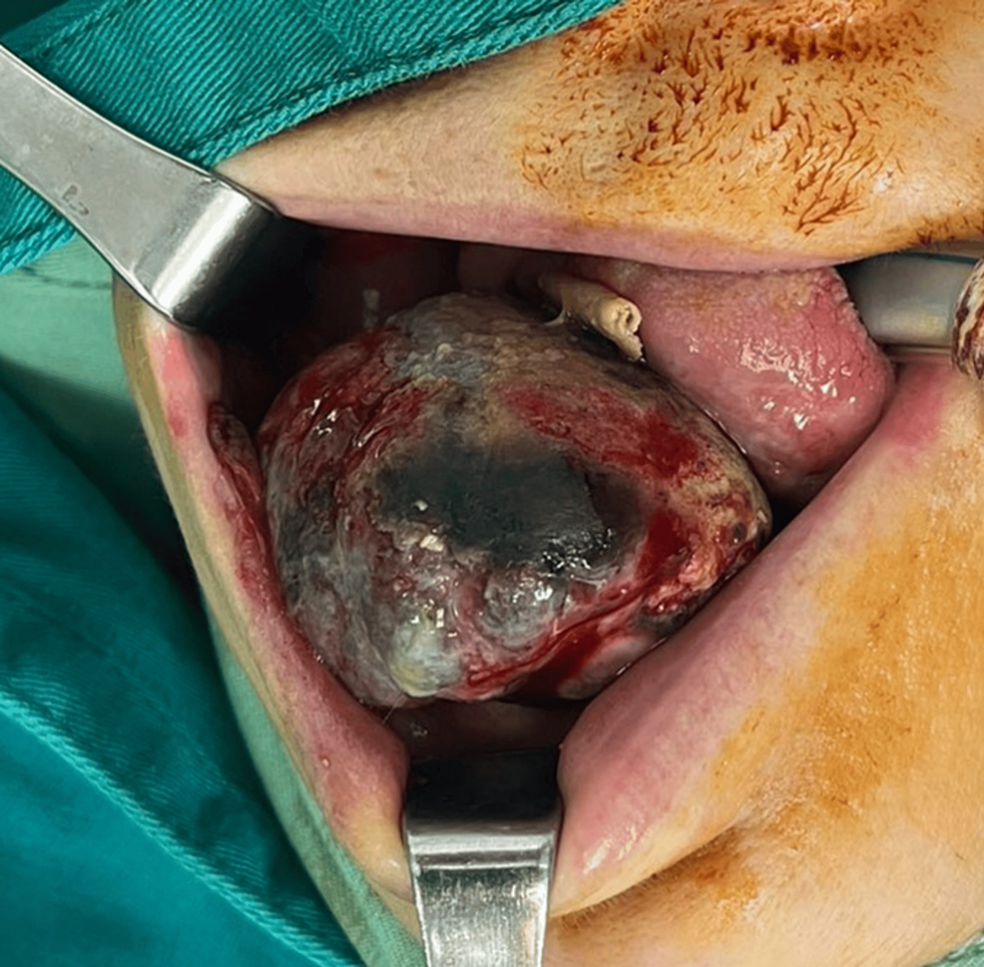
Photograph of the tumor mass present after intubation of the patient, prior to its removal

We present a 59-year-old patient who visited a maxillofacial surgeon in February 2024 due to a tumor mass that was growing in size, progressively filling her oral cavity and making it difficult to speak and eat normally, dating from about two months. The physical examination revealed the presence of a soft-elastic, painless tumor formation with a smooth surface, well-circumscribed margins, and a brown-black color measuring about 8.0x4.0x4.0 cm, located on the gingiva of the lower jaw on the right (Figure 1).

**Figure 2:**
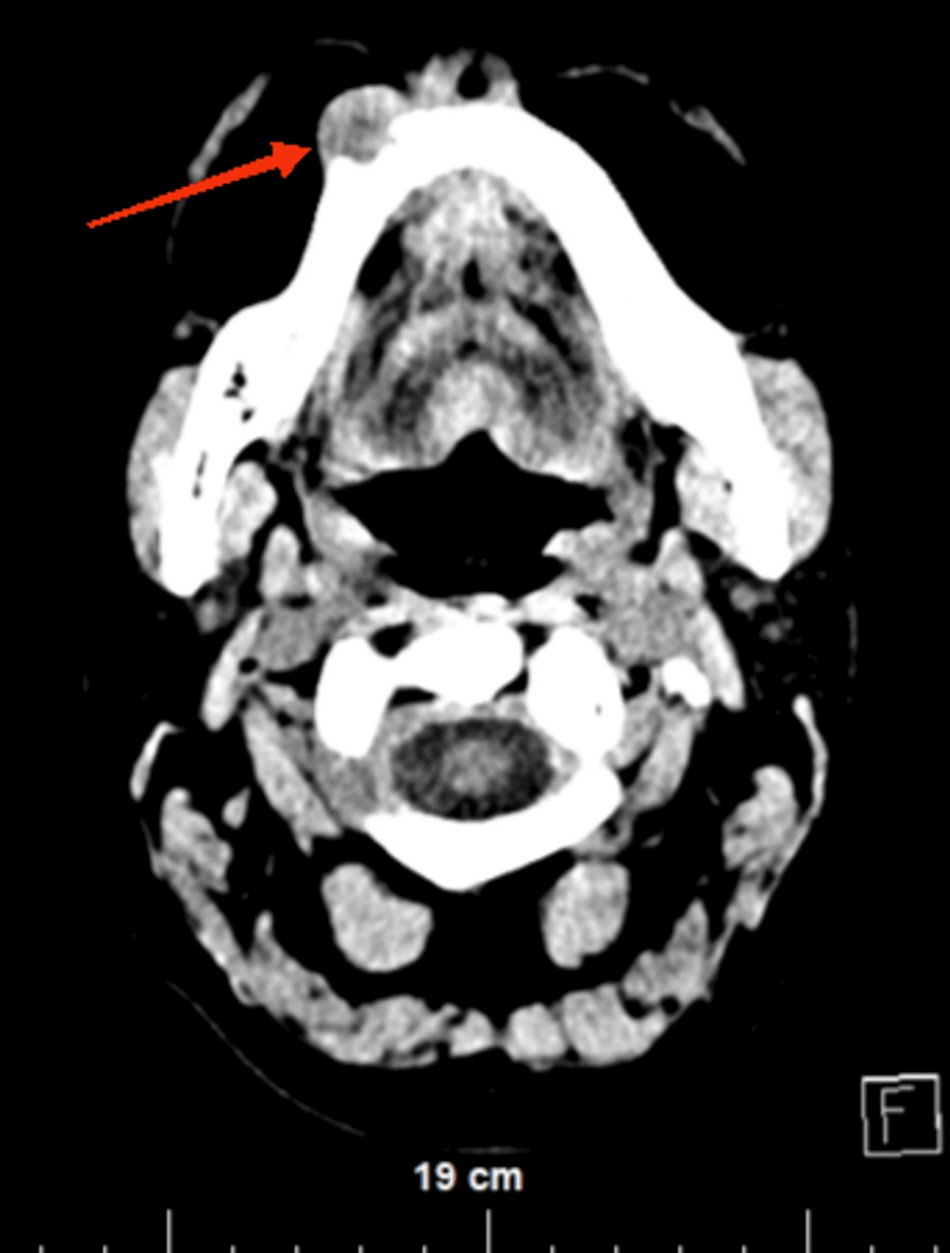
Coronal section from a native CT scan of the head showing the tumor invasion in the alveolar process of the right mandible (red arrow)

**Figure 3:**
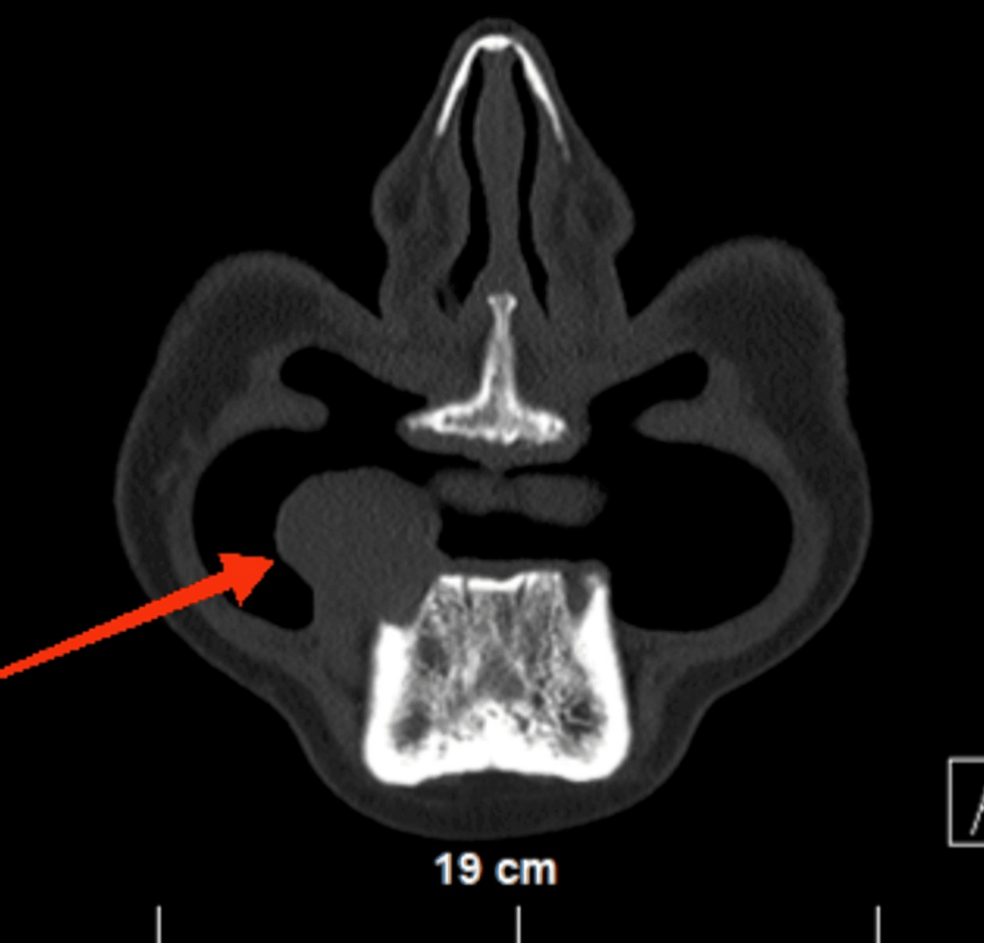
Axial section from a native CT scan of the head showing the tumor invasion in the alveolar process of the right mandible (red arrow)

The patient underwent a CT scan, which visualized the same finding and found the tumor mass to invade the alveolar process of the mandibular body on the right for approximately 1.0 cm (Figure 2 and Figure 3).

The patient had no known accompanying diseases and did not report any known allergies to food or medications. Her family history included breast carcinoma diagnosed in her mother and colorectal carcinoma diagnosed in her grandfather, for which no additional data were available. In 2016, at the age of 51, due to adenocarcinoma of the sigmoid part of the large intestine, the patient was operated on in another medical facility. Due to invasion by the same disease in the uterus and in the left ovary, not only resection of the sigmoid colon but a total hysterectomy was also performed.

Nineteen peritoneal lymph nodes were removed with no data for neoplastic involvement. Two metastatic liver lesions (one in each segment of the liver) were biopsied and histopathological analysis confirmed them to be metastases from adenocarcinoma. Postoperatively, whole-body PET/CT was performed, which revealed secondary involvement of both lobes of the liver, both lungs, the left adrenal gland, and pretracheal and retroperitoneal lymph nodes, with no evidence of persistence and recurrence in the sigmoid colon. By the TNM (Tumour, Node, Metastasis) classification, the carcinoma is staged as T4N0M1 (hepatic (hep), pulmonary (pul), adrenal (adr), lymph nodes (lym)). For the existing metastases, a partial left-sided liver lobectomy and extirpation of the left suprarenal gland were performed together with a partial resection of the left kidney, and in 2017, on the occasion of a mechanical ileus, a partial resection of the ileum was performed.

After the surgical treatment, on the recommendation of the clinical oncologists from the hospital where she was treated, seven courses of chemotherapy with oxaliplatin, 13 courses with capecitabine, six courses of chemotherapy according to the FOLFIRI (folinic acid, fluorouracil, and irinotecan) protocol, two courses of chemotherapy according to the FOLFOX (folinic acid, fluorouracil, and oxaliplatin) 4 protocol and two courses according to the FOLFIRI protocol, targeted therapy with bevacizumab and aflibercept, and immunotherapy with Avastin was prescribed. Since October 2023, she had been on maintenance chemotherapy with capecitabine (1.5 g per day orally), targeted therapy with bevacizumab (10 mg/kg for 14 days intravenously), and immunotherapy with Irinotecan (250 mg/m2 for 14 days intravenously).

**Figure 4:**
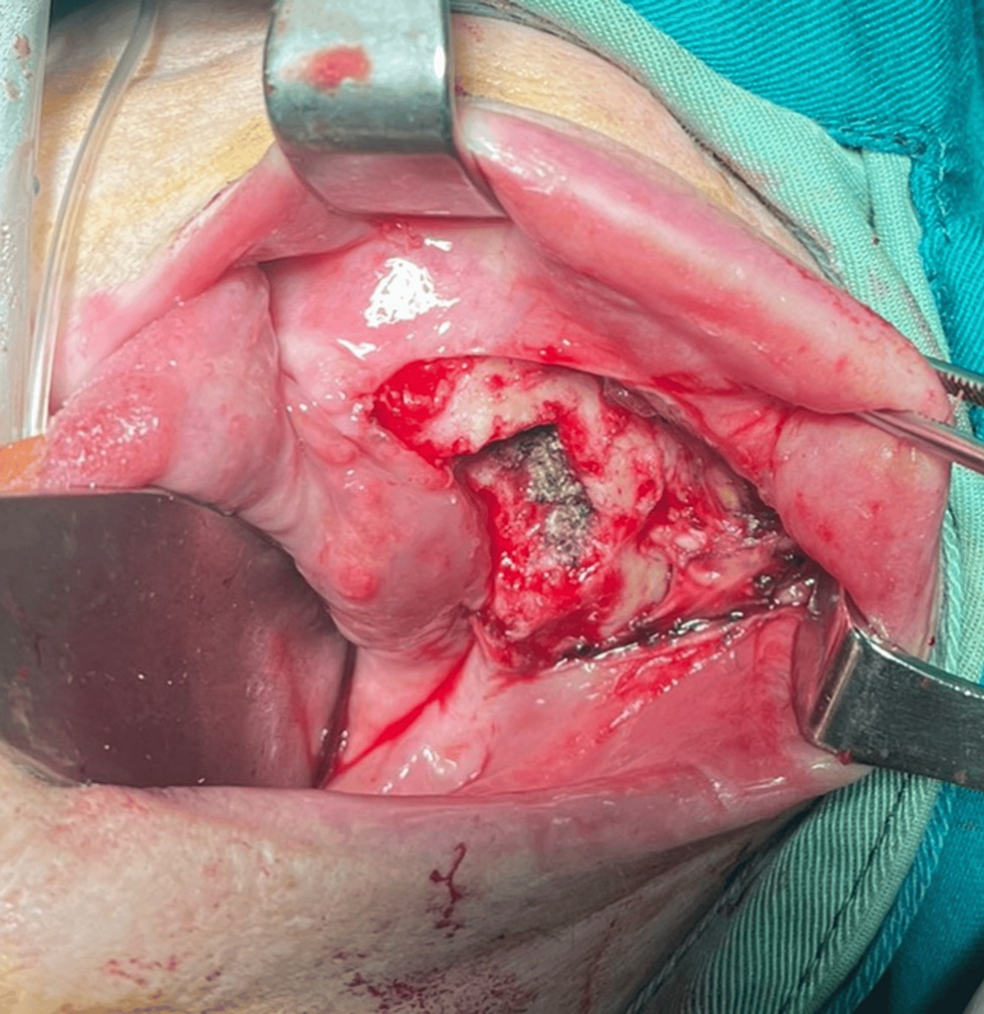
Intraoperative photograph of the bed of the tumor formation after its removal

The patient was hospitalized at the Clinic of Maxillofacial Surgery, University Hospital St. Marina, Varna, Bulgaria, and under general anesthesia with orotracheal intubation, after thorough antiseptic of the operative field and after local anesthetic infiltration with lidocaine and adrenaline in a ratio of 1:100000 for hemostasis, a partial mandibulectomy was performed on the involved alveolar process of the lower jaw on the right along with the adjacent gingiva and tumor mass within clear margins (Figure 4).

**Figure 5:**
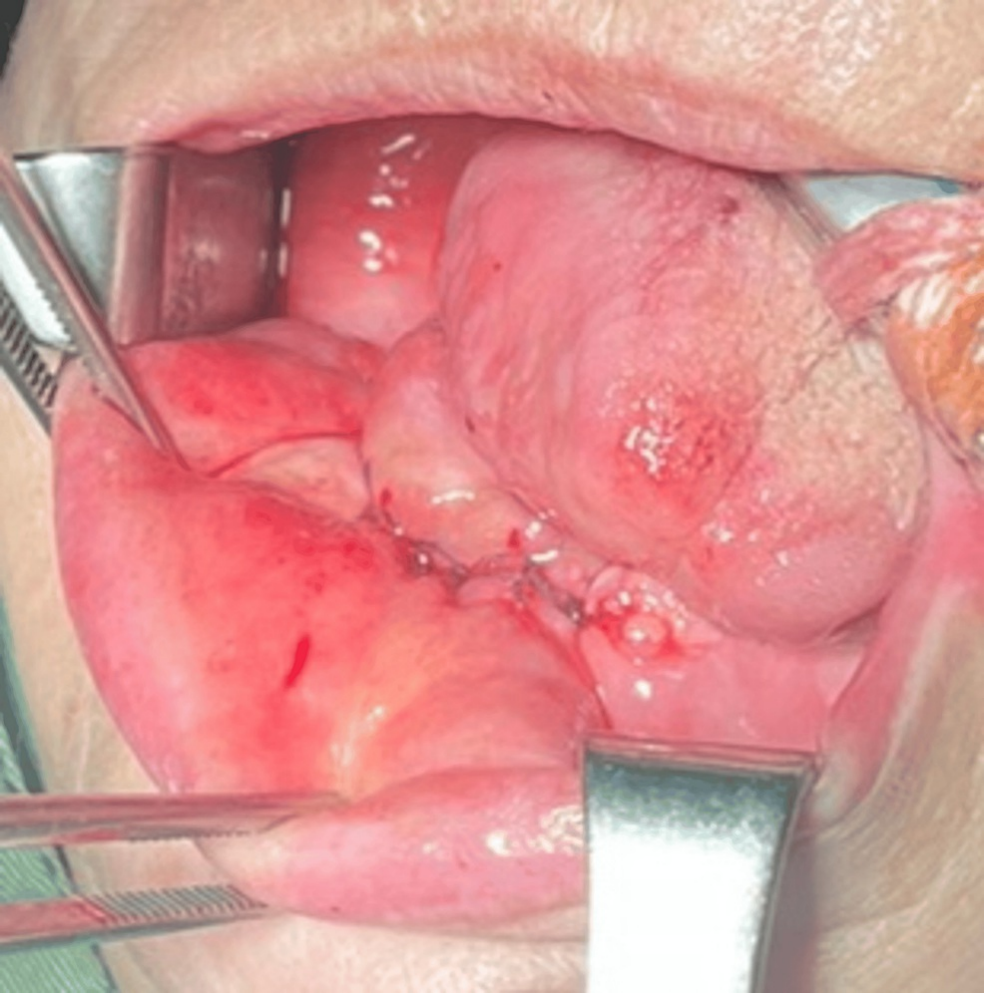
Intraoperative photograph after suturing the soft tissues for the resection of the alveolar process of the lower jaw and the adjacent gingiva and tumor mass

After thorough hemostasis, the soft tissues were sutured with absorbable polyfilament (Figure 5) and the resected material was sent for pathological examination.

**Figure 6:**
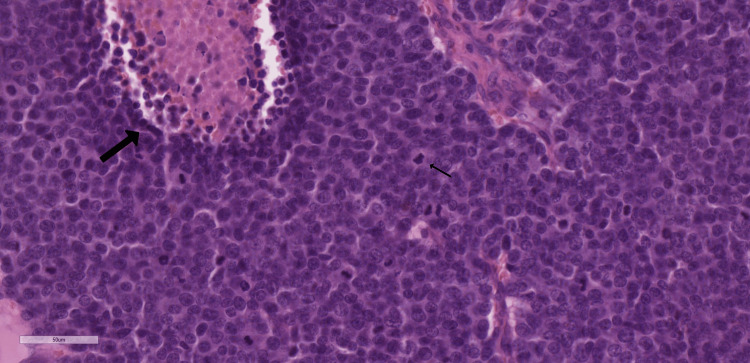
The tumor lesion showing organoid arrangement, enlarged nuclei with prominent nucleoli, fine chromatin, brisk mitotic activity (small arrow), large necrotic areas (large arrow) (H&E, x20)

**Figure 7:**
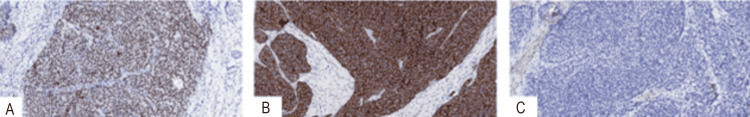
Immunohistochemical evaluation reveals moderate intensity of CDX-2 expression in most cells (A), strong and diffuse positivity for synaptophysin (B), and lack of CK20 staining (C) (IHC, x20)

The histopathological examination revealed a metastatic lesion with morphology of pure LCNEC. Although the metastasis was completely embedded for sampling, there was no evidence of glandular formation. The lesion was strongly and diffusely positive for synaptophysin; most tumor cells showed moderate intensity of CDX-2 expression, while CK20 showed no immunoreactivity at all (Figure 6 and Figure 7). The morphological characteristics and immunoprofile were compatible with metastasis from neuroendocrine carcinoma of gastrointestinal origin.

The sutures were removed on the 10th postoperative day. Until the middle of April 2024, the patient had three follow-up examinations and was without complaints, with a normal postoperative period and calm, normotrophic soft tissues in the area of ​​the surgical intervention. She was directed to the medical facility where she was undergoing chemotherapy and targeted therapy to continue her oncological treatment. In mid-May 2024, after a sharp deterioration in her general condition, she died at home, five months after the appearance of the tumor lesion and three months after its surgical removal. 

## Discussion

Metastases do not always present a clinical picture of malignant tumors, which often leads to their late diagnosis and lack of timely treatment [4,8]. Average survival rate after diagnosis of oral metastases from adenocarcinomas is about six months. Colon carcinomas usually metastasize to the regional lymph nodes, liver, peritoneum, lung, ovary, and rarely to supraclavicular organs [12]. The cases of gingival metastases from colon carcinoma, as in the patient we present, is rare.

A possible mechanism for the occurrence of distant metastases in the lower jaw is the paravertebral Batson venous plexus [13]. The venous systems of the neck, thorax, abdomen, and pelvis are connected by a valveless venous plexus. An increase in pressure in the abdominal cavity can create a venoflow in a cranial direction with the transfer of metastatic cells to the jaws [14]. Such an increase in intra-abdominal pressure can be caused both by a volume-occupying tumor process in the area and by various operative interventions, factors that are present in the case described by us, in which resection of the sigmoid colon, total hysterectomy, lymphatic dissection, liver biopsy, and resection of the ileum was done.

These distant gingival metastases more often affect the lower jaw due to its rich vascularization. The rich capillary network in the gingiva supports the invasion of tumor cells [15], as in the patient we present.

Metastases in the oral cavity can progress rapidly, provoke pain, and bleeding, and/or cause paresthesias [5,16]. In the case of the patient we presented, in just two months, the mass reached a size of up to 8 cm in one of its projections, a process that, in most tumors of other etiology, usually takes years. At an initial stage, metastases affecting the gingiva can easily be confused with reactive or hyperplastic soft tissue reactions [2]. In addition, macroscopically, soft tissue metastases often resemble benign lesions such as pyogenic granuloma, fibroma, giant cell granuloma, and others [7]. In appearance, the lesion we excised had a smooth surface and could easily be confused with these benign lesions in terms of differential diagnosis.

The presence of chronic gingival inflammation is considered to be a prerequisite for the invasion and proliferation of metastatic cells [2]. In this sense, maintaining good oral hygiene would serve as a prevention against gingival metastasis [4].

Gingival metastases can manifest clinically as a dense polypoid tumor, which requires histopathological examination [3]. Adenocarcinoma is found in 65% of cases [3]. With an immunohistochemical examination, we concur whether it is a primary tumor or a metastasis [17].

A histopathological examination, combined with other diagnostic methods, is decisive for the timely diagnosis. Similarities between the primary tumor and the metastatic ones are present and create difficulties in diagnosing [15]. Advances in imaging, molecular profiling, and immunohistochemistry help define the primary focus and treatment options [2]. The final diagnosis is based on the histological examination, clinical examination, and paraclinical examination including imaging studies and immunohistochemical analysis [14]. The current case was diagnostically challenging, as the lesion showed no evidence of glandular morphology in contrast to the primary neoplasm. The tumor exhibited nested growth with high-grade features such as marked nuclear polymorphism and hyperchromasia, nucleolar prominence and “salt and pepper” chromatin, very high mitotic count, and widespread necrosis. The initial impression of neuroendocrine differentiation was supported by strong and diffuse synaptophysin expression. Evaluation of CDX-2 expression in tumor cells was used to verify intestinal origin. Such phenotypical transformation is well described as a treatment-induced event in lung non-small cell carcinoma and prostate adenocarcinoma but is extremely rare in colon adenocarcinoma [18,19]. To our knowledge, this is the second case described in the literature and the first one exhibiting metastatic pattern with oral involvement [20].

Histologically, the differential diagnosis should be made with primary neuroendocrine carcinoma of the oral cavity and metastatic neuroendocrine carcinoma of the lung, breast, or metastatic Merkel cell carcinoma. The positive immunoreactivity for CDX-2 is confirmatory for the intestinal origin and is preclusive of the other organ sites as possible primaries; furthermore, the lack of dot-like paranuclear expression of CK20 is also not compatible for the diagnosis of Merkel cell carcinoma.

Treatment for metastases in the oral cavity is surgical, as in our case, but can also be combined with radiation and chemotherapy. With the current patient, who was already undergoing chemo-, immuno-, and target therapy in another medical facility, after a presentation to the general hospital oncology committee, a decision was made to continue this type of therapy. Radiation treatment is often palliative in case of massive dissemination of the tumor, with the aim of sustaining the quality of life of the patients [5].

## Conclusions

Metastases from adenocarcinomas in the oral cavity are rare but should be considered as a differential diagnosis even in the absence of evidence of a primary lesion, as they may signal a recurrence or primary manifestation of a malignant tumor. In the patient presented by us, the primary tumor and the metastatic disease involving other parts of the body were already known. However, the phenotypical transformation of the primary adenocarcinoma into LCNEC greatly perplexes the differential diagnosis before the immunohistochemical evaluation. The treatment is surgical excision within healthy margins, the goal of which is the complete removal of the gingival lesion. The present clinical case demonstrates the extremely unfavorable prognosis in patients with intraoral secondary involvement, given the short-period lethal outcome after their onset.

## References

[1] Kumar G, Manjunatha B (2013). Metastatic tumors to the jaws and oral cavity. J Oral Maxillofac Pathol.

[2] Hirshberg A, Berger R, Allon I, Kaplan I (2014). Metastatic tumors to the jaws and mouth. Head Neck Pathol.

[3] van der Waal RI, Buter J, van der Waal I (2003). Oral metastases: report of 24 cases. Br J Oral Maxillofac Surg.

[4] Dalirsani Z, Mohtasham N, Samiee N (2020). Metastasis of colon adenocarcinoma to maxillary gingiva and palate. Iran J Otorhinolaryngol.

[5] Hirshberg A, Shnaiderman-Shapiro A, Kaplan I, Berger R (2008). Metastatic tumours to the oral cavity - pathogenesis and analysis of 673 cases. Oral Oncol.

[6] Allon I, Pessing A, Kaplan I, Allon DM, Hirshberg A (2014). Metastatic tumors to the gingiva and the presence of teeth as a contributing factor: a literature analysis. J Periodontol.

[7] Irani S (2016). Metastasis to the oral soft tissues: a review of 412 cases. J Int Soc Prev Community Dent.

[8] Soares AB, Thomaz LA, Duarte MT, de Camargo de Moraes P, de Araújo VC (2011). Metastatic adenocarcinoma of the colon: early manifestation in gingival tissue. Head Neck Pathol.

[9] Kitamura N, Ishida K, Deguchi H, Hata T, Okamoto T, Hosoda M (2007). A case of transverse colon adenocarcinoma metastating to the maxilla and cervical lymph nodes [Article in Japanese]. J Oral Maxil Surg.

[10] Kataoka S, Shibata M, Doi R, Onda M, Sudoh M, Ryoke K (2003). A clinical study of 17 cases of malignant tumors metastatic to the mouth and jaws [Article in Japanese]. J Oral Maxil Surg.

[11] Singh T, Amirtham U, Satheesh CT, Lakshmaiah KC, Suresh TM, Babu KG, Ramachandra C (2011). Floor-of-mouth metastasis in colorectal cancer. Ann Saudi Med.

[12] Cama E, Agostino S, Ricci R, Scarano E (2002). A rare case of metastases to the maxillary sinus from sigmoid colon adenocarcinoma. ORL J Otorhinolaryngol Relat Spec.

[13] Batson OV (1940). The function of the vertebral veins and their role in the spread of metastases. Ann Surg.

[14] de Almeida Lança ML, Carvalho YR, Almeida JD, Kaminagakura E (2023). Hidden colon adenocarcinoma diagnosed from mouth metastasis: case report and literature review. World J Surg Oncol.

[15] Makoto M, Ntege EH, Kazuhide N (2023). Metastatic colon carcinoma in the maxilla: Highlighting the importance of perioperative oral management: a case report. Mol Clin Oncol.

[16] Tomikawa M, Higuchi Y, Saku M, Takeshita M, Yoshida K, Sugimachi K (2001). Carcinoma of the colon metastatic to the lower gingiva. Dig Surg.

[17] Alvarez-Alvarez C, Iglesias-Rodríguez B, Pazo-Irazu S, Delgado-Sánchez-Gracián C (2006). Colonic adenocarcinoma with metastasis to the gingiva. Med Oral Patol Oral Cir Bucal.

[18] Yu HA, Arcila ME, Rekhtman N (2013). Analysis of tumor specimens at the time of acquired resistance to EGFR-TKI therapy in 155 patients with EGFR-mutant lung cancers. Clin Cancer Res.

[19] Zou M, Toivanen R, Mitrofanova A (2017). Transdifferentiation as a mechanism of treatment resistance in a mouse model of castration-resistant prostate cancer. Cancer Discov.

[20] Du F, Han Y, Hu X (2023). Large cell neuroendocrine carcinoma transformation: a novel acquired drug resistance mechanism in colorectal adenocarcinoma. Cancer Innov.

